# Estimation of the effects PM2.5, NO2, O3 pollutants on the health of Shahrekord residents based on AirQ^+^ software during (2012–2018)

**DOI:** 10.1016/j.toxrep.2022.03.045

**Published:** 2022-04-12

**Authors:** Davood Jalili Naghan, Abdolkazem Neisi, Gholamreza Goudarzi, Maryam Dastoorpoor, Abdolmajid Fadaei, Kambiz Ahmadi Angali

**Affiliations:** a Department of Environmental Health Engineering, School of Health, Ahvaz Jundishapur University of Medical Sciences, Ahvaz, Iran; b Environmental Health Department, Air pollution and Respiratory Diseases Research Center, Ahvaz Jundishapur University of Medical Sciences, Ahvaz, Iran; c Department of Biostatistics and Epidemiology, Air pollution and Respiratory Diseases Research Center, Ahvaz Jundishapur University of Medical Sciences, Ahvaz, Iran; d Department of Environmental Health Engineering, School of Health, Shahrekord University of Medical Sciences, Shahrekord, Iran

**Keywords:** AirQ^+^, Health effect, Mortality, Estimation, Shahrekord

## Abstract

**Research objectives:**

Intertwined with modern life, air pollution is not a new phenomenon. Air pollution imposes a significant number of deaths and disease complications on society, and therefore it is very important to determine the extent of its effects on health in any society. This study sought to evaluate the concentration and short-term and long-term excess mortality attributed to PM2.5, NO_2_ and O3 observed in Shahrekord.

**Procedure:**

Hourly concentrations of PM2.5, O_3_, and NO_2_ measured at different stations of the Shahrekord Monitoring Network were obtained from the Shahrekord Department of Environment (DOE). Then, for different air quality monitoring stations, the average 24-hour PM2.5 concentration, the one-hour average NO_2_ concentration and the maximum 8-hour daily O_3_ concentration were calculated using Excel 2010. When the maximum 8-hour daily ozone level exceeds 35, it drops below 35 to calculate the SOMO35 index for modeling.

**Results:**

The death rates of IHD, COPD, lung cancer and ALRI and stroke related to PM2.5 were 176, 7, 0, 10, 105, respectively. The effect of ozone on respiratory mortality was zero. During the study period in Shahrekord, no respiratory mortality was determined due to ozone and acute lower respiratory tract infection (ALRI). this study is first ever study on health effects of air pollution in shahrekord city

**Conclusion:**

A significant number of deaths due to air pollutants in Shahrekord have been reported. It can be concluded that by designing and implementing strategies and measures to control air pollution, both health effects and economic losses are prevented.

## Highlights

• In Shahrekord, no health effects attributed to ozone were identified and with this level of ozone concentration, there is no concern about health outcomes in this city. In Ahvaz, in the last two intervals, health effects attributed to ozone were zero.
• On average, 12%, 7.7%, 13.8%, 4.8% and 12.28% of natural mortality in Shahrekord were due to short-term exposure to PM2.5 in the first, second, third, fourth and fifth years.
• In Shahrekord no natural deaths due to short-term exposure to this pollutant was reported in the sixth and seventh years. These findings are slightly different from those of previous studies.

## Graphical Abstract

**Figure:**
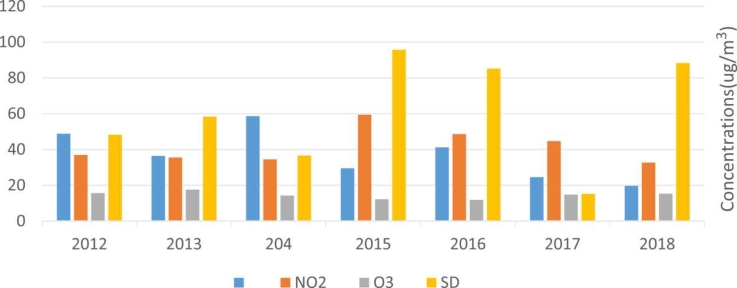

## 1 Introduction

Intertwined with modern life, air pollution is not a new phenomenon. In fact, it is the result of urban emissions resulting from activities such as the production of goods, transportation, heating, recreation and human labor [5], [30]. In addition to environmental degradation and recession, air pollution is one of the top 10 causes of death worldwide, with death rates ranging from 800,000 in 2000 to 1.3 million in 2010 [23]. The effects of air pollution on human health have a wide range, the most common of which include respiratory and cardiovascular diseases [4], [20], [32], [33].

Natural processes absorb pollutants that cause air pollution to some extent and restore air quality. However, as the limitations are exceeded, pollutants accumulate in the environment and air quality deteriorates. According to WHO statistics and data in 2006, more than 80% of urban residents are exposed to air quality levels that go beyond the WHO guidelines (WHO, [32]).

Today, many cities in Iran face poor air quality and dust [7], [9], [11], [13], [14], [15], [16], [26], [36].

Numerous instruments of varying complexity are developed and used to estimate the impact of public health resulting from changes in air quality (which includes both premature deaths and related diseases and their associated economic value) [21], [17], [10], [28], [1]. Among these tools AirQ^+^ software (WHO and US Environmental Protection Agency (EPA) and the Environmental Benefits Mapping and Analysis Program (BenMAP - CE), are widely used.

The AirQ ^+^ model provided by the World Health Organization (WHO) is the most authoritative tool for assessing the adverse effects of exposure to air pollution on human health. The software uses data processed by Excel to estimate the relative risk of the accident and the attributable component, and displays the result as illness and mortality [8], [12].

Because air pollution imposes a significant number of deaths and morbidities on society, it is important to determine the extent of its effects on health in any society. In addition to health-related goals, these results provide a rationale for lawmakers and officials to set new air pollution standards to increase funding for strategies and measures to reduce air pollution [22], [29], [19].

Management programs to control air pollution in large cities are considered as the most important strategies. They use accurate sources of data and information about environmental conditions to determine all the effects of air pollution on human health [24,2].

Assessing the effects of air pollution on health cannot only determine the adverse effects of air quality on public health. However, it will also be useful when considering the potential implementation of various air quality policies. Therefore, assessing potential health effects is a reliable point for public health and environmental professionals. The present study aims to evaluate the concentration and short-term and long-term health effects attributed to standard pollutants on residents of Shahrekord (Iran) from 2012 to 2018.

## 2 Procedure

### 2.1 Study, course and estimation method

With an area of 70 square kilometers, Shahrekord is the center of Chaharmahal and Bakhtiari Province in Iran. The geographical coordinates of Shahrekord are 49º22 / E and 32º20 / N in the plains of Chaharmahal and Bakhtiari Province.

This is an ecological study whose main purpose was to Assessment of the effects PM2.5, NO2, O3 pollutants on the health of Shahrekord residents based on AirQ + software during

Sampling was performed from March 2012 to March 2018.

The total population of Shahrekord in the first, second, third, fourth, fifth, sixth and seventh period was 82,450, 95,632, 132,450, 170,198, 222,198, 275,549, and 288,199, respectively.

Due to paucity of comprehensive studies in Iran and the lack of sufficient information on the health consequences attributed to pollutants, new findings of meta-analysis studies obtained by AirQ^+^ software in other countries were used in the present study, and this was one of its major limitations. The health consequences discussed included the following: natural mortality in the population over 30 years of age, mortality from (ALRI) (Acute Lower Respiratory Infections) in people under 5 years of age, (COPD) (Chronic Obstructive Pulmonary Disease), heart disease, lung cancer in the population over 30 years of age, and stroke and (IHD) (Ischemic Heart Disease), in the population over 25 years.

The Indicators used in this study are presented in Table 1, Table 2.

**Table 1:** The health outcomes and at-risk population during the study period.

Pollutant	Health outcome	At-risk population						
		2012	2013	2014	2015	2016	2017	2018
PM_2.5_	Natural mortality	55,200	72,425	85,710	104,642	115,942	143,830	178,730
	ALRI	4570	6991	9351	12,642	16,835	20,876	25,885
							
	**COPD mortality**	55,200	72,425	85,710	104,642	115,942	143,830	178,730
	LC mortality	55,200	72,425	85,710	104,642	115,942	143,830	178,730
	IHD mortality	60,800	76,425	89,710	104,642	120,942	139,830	174,350
	Stroke mortality	60,800	76,425	89,710	106,642	120,942	139,830	174,350
NO_2_	Natural mortality	55,200	72,425	85,710	104,642	115,942	143,830	178,730
**O**_**3**_	Respiratory mortality	55,200	72,425	85,710	104,642	115,942	143,830	178,730

**2012**(2012–2013) **2013**(2013–2014) **2014**(2014–2015) **2015**(2015–2016) **2016**(2016–2017) **2017**(2017–2018) **2018**(2018–2019). natural mortality for adults > 30 years old; Acute lower respiratory infection for children 5 years; Chronic obstructive pulmonary disease mortality for adults > 30 years; lung cancer mortality for adults > 30 years; Ischemic heart disease mortality for adults > 25 years; Stroke mortality for adults > 25; All natural mortality for adults > 30 years old; Respiratory mortality for adults > 30 years old.

**Table 2:** The health outcomes and baseline incidence during the study period.

Pollutant	Health outcome	Baseline incidence (per 100,000)						
		2012	2013	2014	2015	2016	2017	2018
PM_2.5_	Natural mortality	1091	1024	1001	987	1010	980	995
	ALRI	10	10	12	11	12	10	13
	**COPD mortality**	9	4.1	5.9	5.5	4.1	4.8	4.1
	LC mortality	12	11.7	9.5	8	8.8	12.1	16.1
	IHD mortality	165	161	115	135	104	118.6	221
	Stroke mortality	62	60	85	99	113	120	121
NO_2_	total mortality	1081	1029	1011	975	1020	985	997
**O**_**3**_	Respiratory mortality	6	8.3	11.1	12.1	9.5	11.8	10.2

In this study, we tried to estimate the impact assessment criteria for all pollutants. However, due to the fact that in this software, the effects of carbon monoxide and sulfur dioxide pollutants could not be calculated from the standard pollutants, unfortunately, it has not been possible to estimate the health effects of these two pollutants.

### 2.2 Air quality data

This study was a time series and ecological research. Data on hospital admissions, total mortality, and mortality from cardiovascular and respiratory diseases from 2012 to 2018 (7 years) were collected on a daily basis from the main and referral hospitals in shahrekord and also from the health deputy of shahrekord University of Medical Sciences.

Daily concentrations of pollutants were collected on a daily basis from the Department of Environmental Protection(DOE) of chahar mahal and bakhtiari provinces for 7 years. These data included ozone, nitrogen dioxide and particles smaller than or equal to 2.5 µm that were measured in three monitoring stations installed in stations of the Jahad Square, Ostandari Square, and chaharmahal Square in shahrekord. In these stations, outdoor air pollutants and particles smaller than or equal to 2.5 µm were measured separately in different ways. To measure suspended particles, air is first pumped into the measuring devices. The device then measures the particle concentration based on the intensity of adsorption and records it every one hour. In this method, stations that have 75% of the complete data are selected according to Aphekom and WHO methods [10], [28].

The location of sampling stations was selected based on crowded places in terms of vehicle traffic and population.

The method for determining the average concentration of each pollutant was as follows.

PM2.5: the 24-h average (24-h).

NO_2_: the maximum average of 1 h.

O_3_: the total ozone i.e., more than 35 ppb (SOMO35) [3].

For PM2.5, the 24-hour average for each station was first calculated and then, using the 24-hour average of different stations, the 24-hour average of the whole city was calculated. For NO2, in order to calculate the maximum of 1 h, the average was taken only from the days that had data for at least 18 h (0.75) and finally, the maximum hourly concentration for each day was selected and considered as the average of that day. To calculate SOMO35, an average concentration of a maximum of 8 h per day was calculated in ppb for all stations, which is attributable to SOMO35. That is, values below 35 ppb were not considered to have health effects.

(1) $$SOMO35=\frac{\sum_{i}\max\left\{0,C_{i}-35ppb\right\}}{SOMO35_{uncorrected}}\cdot \frac{N_{total}}{N_{valid}}$$

where SOMO35_uncorrected_ is uncorrected form of SOMO35. N_total_ is the total number of days in a year, and N_valid_ is the number of days for which valid data is available.

Air pollution standards for PM2.5, NO2 and O3 are set by the World Health Organization at 10, 40 and 100 micrograms per cubic meter, respectively (average annual concentration) [35].

### 2.3 Demographic information

In Iran, population statistics are monitored and recorded daily by health centers under the auspices of medical universities, and thus it is the most reliable source of population statistics. The statistics used in this study were annually collected from health centers under the auspices of shahrekord University of Medical Sciences.

### 2.4 Baseline incidence

The rate of baseline incidence (BI) related to each health effect (number of basic health consequences per population (100,000)) for each city was obtained separately from the Deputy of Health of Shahrekord University of Medical Sciences and Ministry of Health.

BI is obtained according to the following formula:

(2) BI = AP × B

where B is the initial rate of beneficial health outcome per 100,000 people, and AP is the attributable ratio (AP) of the health effects of air pollution·

### 2.5 Relative risk (RRs)

In this study, due to limitations such as insufficient previous studies determining the RR index for the target areas, the default values of relative RR risk in the AirQ ^+^ model were used for this study. These values are obtained from meta-analysis studies [27]. In this study, RR values for the total mortality and respiratory mortality were 1.065 (1.04–1.083) and 1.014 (1.005–1.024), respectively. The rate of ALRI, COPD, lung cancer, IHD, and stroke were also estimated using IER.

The relative risk index for different concentrations was obtained according to the following formula [27].

(3) $$\mathrm{RR}\left(X\right)=e^{\beta (X-X_{0})}$$

X, X0 and β on average represent the concentration of a contaminant in the target city, the amount of cut and the change in RR for a unit of change in concentration X.

### 2.6 Statistical analyses

The concentrations of all three pollutants were analyzed separately in different years. Since the data had normal distribution and equal variance, Kolmogorov-Smirnov and Levene test (equality of variance and normal distribution) and Kruskal-Wallis test (inequality of variance) were used.

## 3 Results and discussion

In this study, the health effects attributed to PM2.5 and NO2 for concentrations higher than those set by WHO guidelines and those attributed to O3 concentrations above 35 ppb were estimated by AirQ ^+^ software.

To interpret the results correctly, it should be noted that the health effects attributed to PM2.5 and NO2 were calculated only for concentrations higher than the WHO guidelines. In addition, the health effects of O3 were estimated for concentrations above 35 ppb.

The mean seven-year concentrations of PM2.5, NO2 and O3 were 49.94 (287.48%) and 59.42, respectively. SOMO35 ozone levels were zero in all years of the study period.

**Fig. 1:**
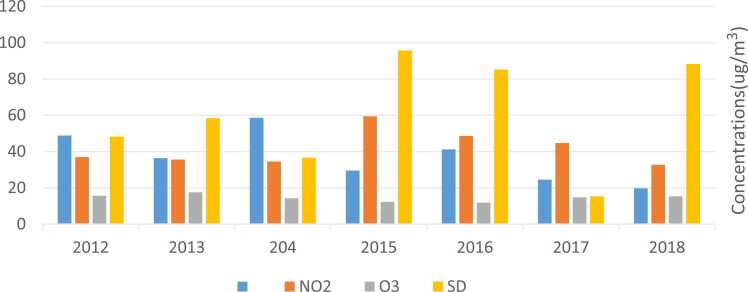
Annual averages and standard deviations of the PM_2.5_, NO_2_, and O_3_ during the study period in ambient air of Shahrekord City.

The annual average and standard deviation of pollutants are shown in Fig. 1.

The average annual concentrations of PM2.5 pollutants during the years 2012–2013, 2013–2014, 2014–2015, 2015–2016, 2016–2017, 2017–2018 and 2018–2019 are 48.8, 36.37, 58.6, 29.5, 41.2, 24.5 and 19.7, respectively. This was 4.8, 3.63, 5.86, 2.95, 4.12, 2.45, and 1.97 times the amount of the WHO guideline (10 micrograms per cubic meter) [35]. These results are consistent with other studies [18].

The average annual concentrations of NO2 pollutants during the years 2012–2013, 2013–2014, 2014–2015, 2015–2016, 2016–2017, 2017–2018 and 2018–2019 are 36.9, 35.50, 34.50, 59.42, 48.6, 44.7 and 32.7, respectively. This was higher only in the period 2014–2015 and less in the rest of the periods than the guidelines of the World Health Organization (40 micrograms per cubic meter).

The average annual concentrations of O_3_ pollutants during the years 2012–2013, 2013–2014, 2014–2015, 2015–2016, 2016–2017, 2017–2018 and 2018–2019 are 15.6, 17.56, 14.22, 12.23, 11.85, 14.75 and 15.3, respectively. For all years, it has been less than the guideline of the WHO (100 micrograms per cubic meter) [35] (21). These results are consistent with other studies.

The reason for the lack of effect of ozone on the health of the inhabitants of this area was the low amount of ozone (less than the allowable limit) in the air of Shahrekord, which had no adverse effect.

In this study, it was found that the trend of pollutant concentrations in Shahrekord during the study period was irregular and did not follow a specific rule.

Dastoorpoor et al. [6] examined cardiovascular mortality attributable to ambient air pollutants in Ahvaz, Iran, and found that the average daily concentrations of ozone and nitrogen dioxide in the period 2008–2015 were 62 (31.07 ppb) and 44.20 micrograms per cubic meter [6].

Table 3, Table 4 attributable ratio (AP) and number of natural and respiratory deaths due to short-term contact and Table 6, Table 5 attributable ratio (AP) and number of natural and respiratory deaths due to long-term contact with PM2.5, NO2 and O3. The attribution ratio represents the percentage of health outcomes in a population due to a given pollutant. In the case of natural death, the highest and lowest natural mortality were related to NO_2_ and O_3_, respectively. In general, no ozone-related health effects have been identified, and with this ozone concentration, there is no concern about the occurrence of health-related consequences. The highest long-term health effects of PM2.5 and NO2 were observed in the third and sixth years, respectively.

**Table 3:** Attributable proportion due to short-term exposure to PM_2.5_, NO_2_, and O_3_ during the study period.

Pollutant	Health outcome	Attributable proportion (%)						
		2012	2013	2014	2015	2016	2017	2018
PM_2.5_	Natural mortality	2.87	1.38	4.02	0.55	1.96	0	0
NO_2_	Natural mortality	0.72	0.69	0.66	1.32	1.04	0.93	0.61
**O**_**3**_	Natural mortality	0	0	0	0	0	0	0
	Respiratory mortality	0	0	0	0	0	0	0
	cardiovascolar mortality	0	0	0	0	0	0	0

**2012**(2012–2013) **2013**(2013–2014) **2014**(2014–2015) **2015**(2015–2016) **2016**(2016–2017) **2017**(2017–2018) **2018**(2018–2019).

**Table 4:** Attributable cases due to short-term exposure to PM_2.5_, NO_2_, and O_3_ during the study period.

Pollutant	Health outcome	Attributable cases						
		2012	2013	2014	2015	2016	2017	2018
PM_2.5_	Natural mortality	16	10	35	6	28	0	0
NO_2_	Natural mortality	**6**	**7**	**0**	**22**	**23**	**25**	**17**
**O**_**3**_	Natural mortality	0	0	0	0	0	0	0
	Respiratory mortality	0	0	0	0	0	0	0
	cardiovascolar mortality	0	0	0	0	0	0	0

**2012**(2012–2013) **2013**(2013–2014) **2014**(2014–2015) **2015**(2015–2016) **2016**(2016–2017) **2017**(2017–2018) **2018**(2018–2019).

**Table 5:** Attributable proportion due to long-term exposure to PM2.5, NO2, and O3 during the study period.

Pollutant	Health outcome	Attributable proportion (%)						
		2012	2013	2014	2015	2016	2017	2018
PM_2.5_	Natural mortality	20.82	14.67	25.35	11.07	17.11	8.35	5.67
	ALRI	24.62	19.23	28.09	28.97	21.05	21.5	9.05
	COPD mortality	20.41	16.04	23.27	13.11	17.86	10.64	7.86
	LC mortality	18.01	13.65	20.97	10.85	15.44	8.57	6.14
	IHD mortality	21.40	17.61	23.85	14.85	19.25	12.39	9.48
	Stroke	19.34	15.47	21.82	12.82	17.09	10.52	7.9
NO_2_	Natural mortality	10.25	9.74	9.38	18.01	14.33	13.01	8.72
**O**_**3**_	Respiratory mortality	0	0	0	0	0	0	0

**Table 6:** Attributable cases due to short-term exposure to PM_2.5_, NO_2_, and O_3_ during the study period.

Pollutant	Health outcome	Attributable cases						
		2012	2013	2014	2015	2016	2017	2018
PM_2.5_	Natural mortality	117	109	217	117	200	118	101
	ALRI	0	0	0	0	0	0	0
	COPD mortality	1	1	1	1	1	1	1
	LC mortality	1	1	1	1	1	1	1
	IHD mortality	23	22	25	21	24	22	39
	Stroke	7	8	17	13	23	19	18
NO_2_	Natural mortality	86	95	124	303	322	351	250
**O**_**3**_	Respiratory mortality	0	0	0	0	0	0	0

**2012**(2012–2013) **2013**(2013–2014) **2014**(2014–2015) **2015**(2015–2016) **2016**(2016–2017) **2017**(2017–2018) **2018**(2018–2019).

On average, natural mortality in Shahrekord due to short-term exposure to PM2.5 in the first, second, third, fourth, fourth and fifth years was 12%, 7.7%, 13.8%, 4.8% and 12.28% respectively. In the sixth and seventh years, there were no natural deaths due to short-term exposure. These findings are slightly different from previous studies.

For example, Hopke et al. reported that about 3.60–5.02% of natural deaths in Ahvaz were due to exposure to PM2.5 [18].

The total number of natural mortality due to long-term contact with PM2.5 was 1278 and short-term contact was 95. The number of natural mortality due to long-term exposure to NO2 was 1531 and short-term exposure was 100. NO_2_ had a worse status than PM2.5 and was identified as a responsible pollutant. No deaths were observed due to ozone.

In a study conducted by Naddafi et al. in Tehran (Iran) entitled "Determining the effects of air pollutants in Tehran in 2012 on health", the results showed that the largest share of health effects attributed to particulate matter was 2.5 and 10 µm [25]. While during the study period, the most effects were attributed to NO_2_.

In a study in Ahvaz, Iran AP and the total number of respiratory deaths due to ozone exposure were determined to be 6.17% and 173, respectively, which is higher than the present study. This difference may be due to a numerical difference in geographical distribution, population at risk (recording the population census, taking into account growth rate), or a difference in the average daily recording of pollutants.

The death rates of IHD, COPD, lung cancer and ALRI and stroke related to PM2.5 were 176, 7, 0, 10, 105, respectively. The effect of ozone on respiratory mortality was zero. During the study period in Shahrekord, no respiratory mortality due to ozone and acute lower respiratory tract infection (ALRI) were determined.

No studies have been performed to evaluate the health effects of short and long-term exposure to gaseous pollutants in Shahrekord. This study is investigate only, the health effects of pollutants in Shahrekord.

Air pollution imposes direct and indirect costs on countries. The World Bank Group report shows that the health effects of ambient air pollution in Iran lead to a decrease in total welfare and GDP of $ 30.6 million and 2.48% in 2013, respectively. In addition, the total lost labor force production and its share in Iran's GDP was $ 1471 million and 0.12%, respectively [31]. Therefore, air pollution in Shahrekord is considered as a serious problem and requires the attention of policy makers to take preventive and control measures in this regard. By designing and implementing strategies and measures to control air pollution, including continuous monitoring of air pollution indicators and identifying influential factors, we can prevent both health effects and economic losses caused by air pollution. Since motor vehicles and dust storms in the Middle East are the main source of air pollution in Shahrekord, to reduce the effects of these pollutions in Shahrekord, program development and cooperation between organizations and even neighboring countries as well as improvements in motor vehicles will definitely lead to significant effects.The results of this study will be useful if accompanied by political and economic regulations.

The strengths of this study were: **First**, all data related to air pollutants and the baseline incidence have been collected from government and trusted organizations from the center in Chaharmahal and Bakhtiari Province (Shahrekord) and the Ministry of Health, which has led to a relatively large volume of data. **Secondly**, compared to previous studies, given the length of the 7-year period and the large amount of data, it allowed us to examine communications at a high level of reliability and reliability. **Third**, the inclusion of 4 air pollution monitoring sites has provided a basis for better demonstration of the effects of air pollution compared to other studies.

However, this study has its limitations. Our research has some limitations. Similar to other studies, the effects and interplay between air pollutants and health (mortality and hospital admission) requires interpretation - the effect might vary for each region. **Secondly**, fixed monitoring stations in specific urban locations represent the total exposure of people to pollutants for all residents of the area. This generalization may not always be true and represent the total contact of the population with the pollutant. On the other hand, exposure to pollutants depends on many conditions, such as indoor and outdoor activities, residence, occupational exposure, and so on.

**Third**, other potential contributing factors, such as BMI, education and income, smoking, physical activity, and medical history, are not included in this study, which may have a potential impact on the relationship between air pollutants and hospitalization, mortality etc.

**Fourth**, the relative risks (RR) used to estimate the effects of pollutants on health are based on program defaults and studies in other countries.

**Finally**, the main limitation of this study is its ecological nature, which does not allow us to control potentially disruptive factors at the individual level, such as respiratory habits, age, diet, existing or genetic diseases, socioeconomic status etc.

## 4 Conclusion

We investigated the short-term and long-term health effects associated with exposure to PM2.5, NO2 and O3 in Shahrekord over a seven-year period using the AirQ + model. Contaminant concentrations were often higher than WHO guidelines. The short-term effects of PM2.5 on health were greater than those of NO2. However, the long-term effects of NO2 were greater than PM2.5 and overall, the effects of NO2 were greater than PM2.5. The health effects of ozone were zero. In general, all mortality is estimated to be due to PM2.5 and NO2.

The total number of natural deaths due to PM2.5 and NO2 in all studied years was 1074 and 1631, respectively. The total number of deaths from IHD, COPD, lung cancer, ALRI and stroke attributed to PM2.5 were 176, 7, 10, 0, 105, respectively. Other studies have shown that the health effects of air pollution impose direct and indirect costs on countries. Therefore, by designing and implementing strategies and measures to control air pollution, we might be able to curb both health effects and economic losses. Policymakers and experts in the field should focus on reducing air pollution.

Therefore, due to the worrying situation of pollutants in Shahrekord, the relevant authorities are advised to take preventive and corrective measures to reduce or eliminate pollutants while conducting the necessary research in this field.

## Declaration of Competing Interest

The authors declare that they have no known competing financial interests or personal relationships that could have appeared to influence the work reported in this paper.
